# Supplementary material for: Molecular Assessment of HER2 to Identify Signatures Associated with Therapy Response in HER2-Positive Breast Cancer
Source: Cancers (Basel). 2022 Jun 4;14(11):2795. doi: 10.3390/cancers14112795 (PMC9179327; doi:10.3390/cancers14112795)
Supplement: Supplementary file 1 [file cancers-14-02795-s001.zip › cancers-1726253-supplementary.pdf]

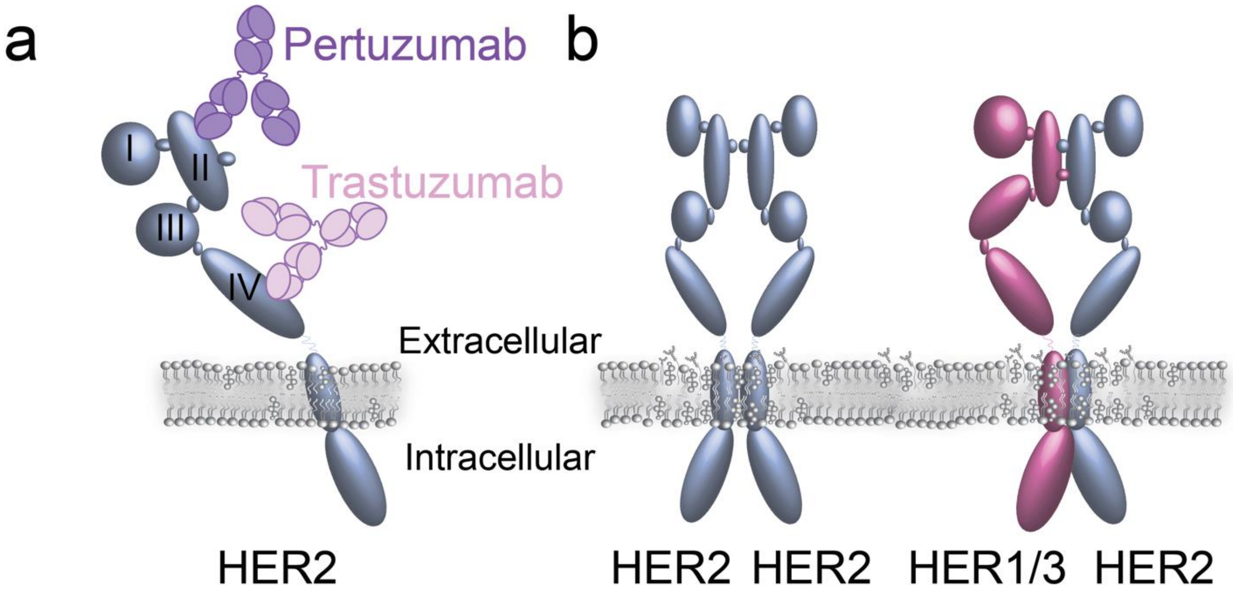

Figure S1. Schematic representation of HER2. **a.** Scheme of trastuzumab and pertuzumab binding to HER2. **b.** Schematic representation of HER2 homodimers and heterodimers.

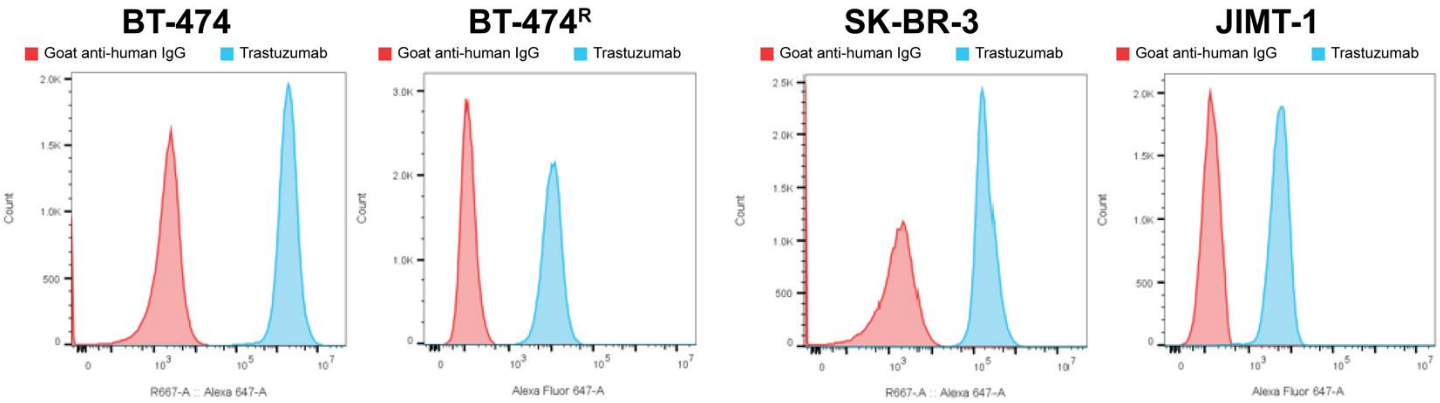

Figure S2. Cultured cell lines express HER2 protein. HER2 protein expression was detected in BT-474, BT-474[R], SK-BR-3, and JIMT-1 cells by flow cytometry using trastuzumab-AF647.

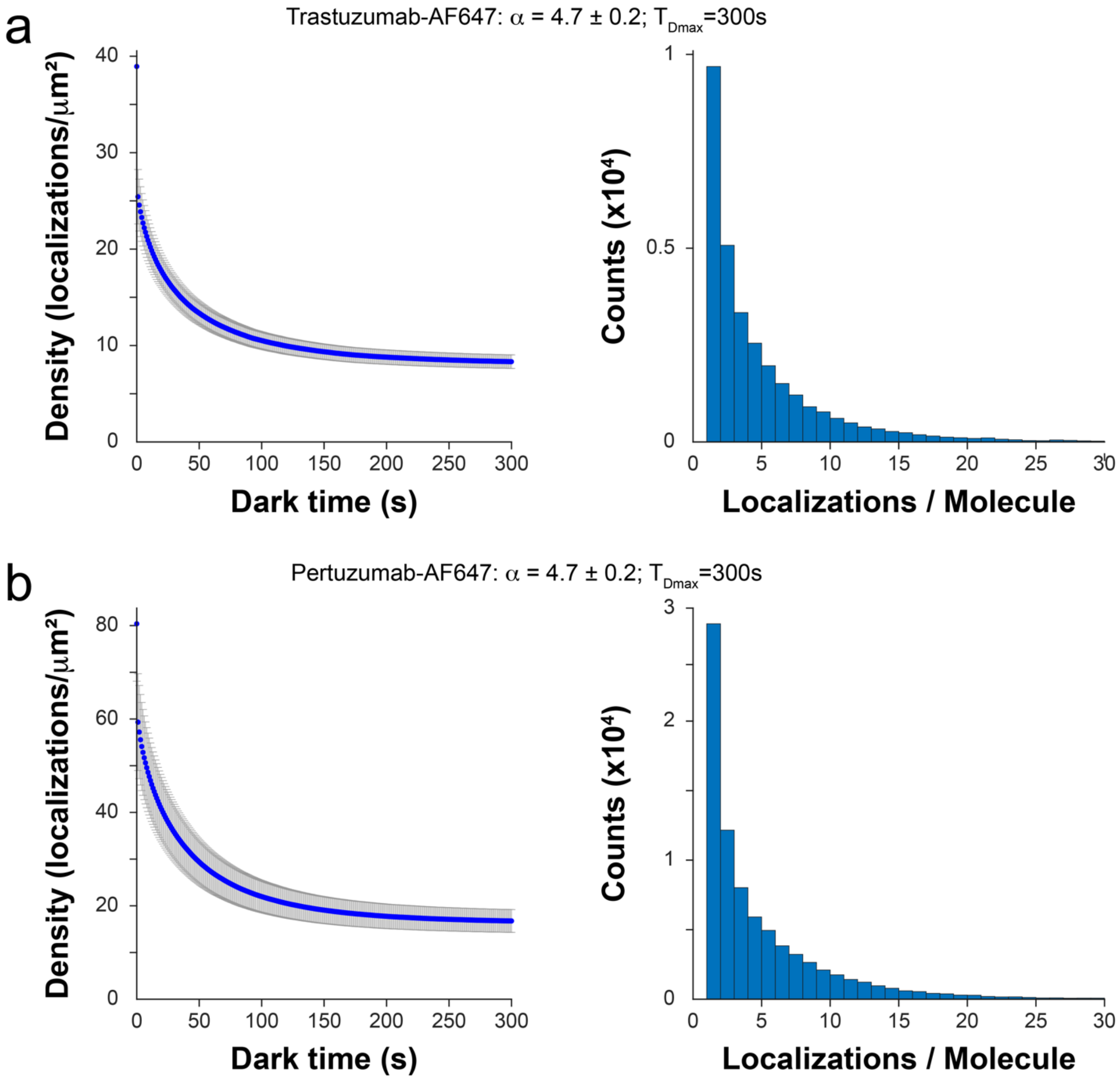

Figure S3. Photophysical properties of fluorescent reporters. **a.** Average number of localizations (α) for trastuzumab-AF647 was 5 and the maximum dark time (T_{Dmax}) was 300 s**.** **b.** Average number of localizations for pertuzumab-AF647 was 5 and the maximum dark time was 300 s. Localizations were detected with a 64 μm^2 region of interest (ROI); 10 ROIs were assessed for trastuzumab-AF647 and 13 ROIs were assessed for pertuzumab-AF647.

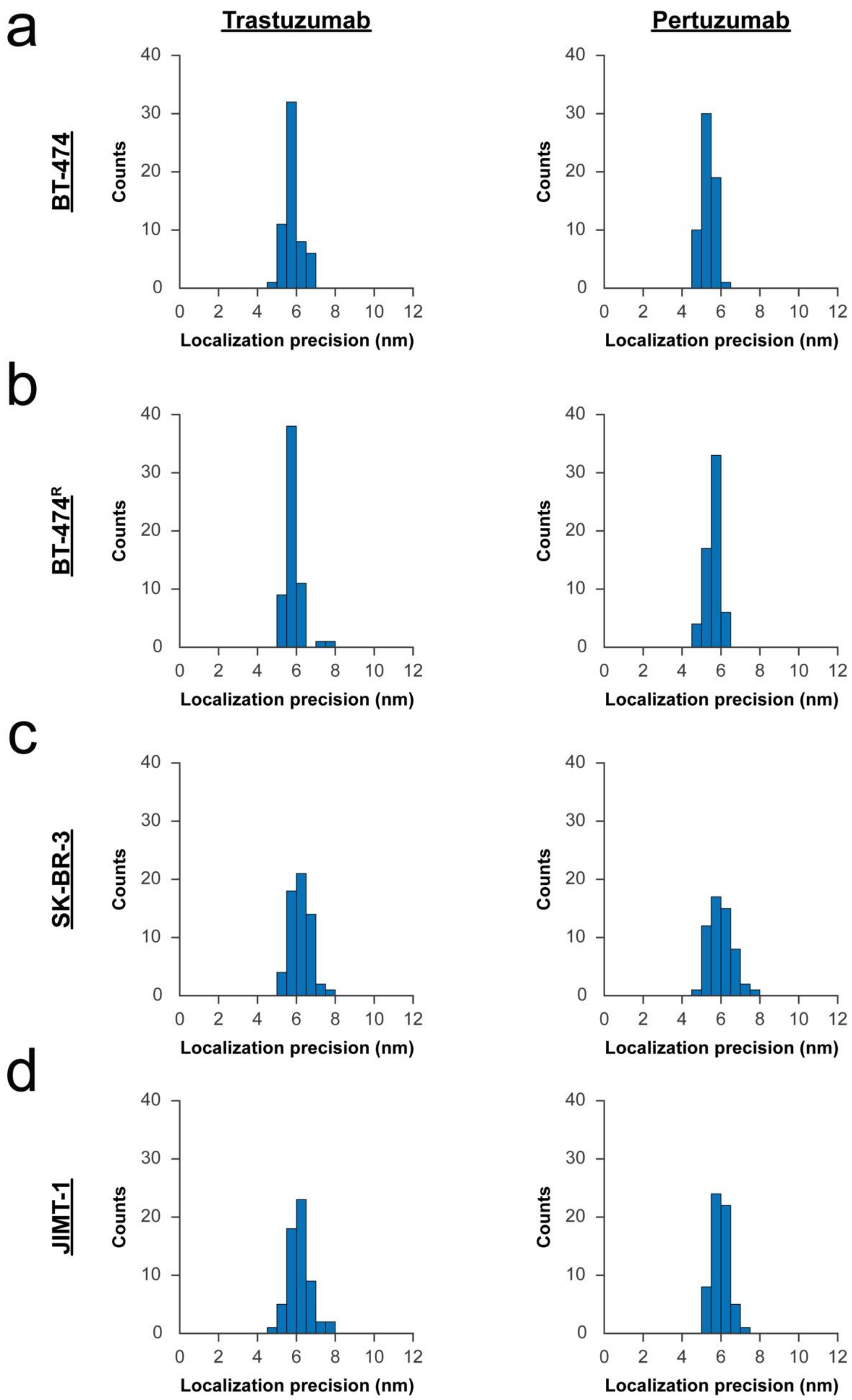

Figure S4. Distributions of lateral localization precisions in breast cancer cell lines. Lateral localization precisions (σ) were obtained using NIS Elements software for all investigated ROIs in cultured cell lines for detection of HER2 with either trastuzumab-AF647 (left) or pertuzumab-AF647 (right). Distributions of σ are shown for: **a**. BT-474 cells, **b**. BT-474^{R} cells, **c**. SK-BR-3 cells, and **d**. JIMT-1 cells. .

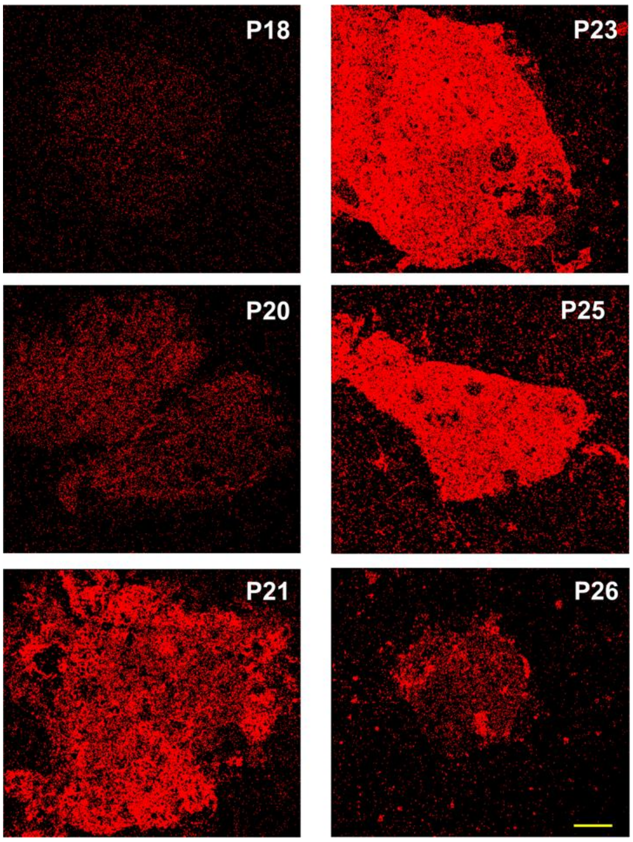

Figure S5. Representative SMLM images of HER2 for individual patients. Scale bar is 5 μm.

Table S1 Summary of qSMLM data for cultured cell lines.

a	Trastuzumab-AF647		Pertuzumab-AF647	
	BT-474	BT-474[R]	BT-474	BT-474[R]
**Density (molecules/µm²)**				
Mean ± SEM	221 ± 12	182 ± 7	85 ± 3	92 ± 4
Median	195	179	83.8	87.1
CV (%)	43	27.6	28.9	31.9
Skewness	1.4	-0.14	0.29	1.4
Kurtosis	1.6	-1.1	-1.2	2.4
$P\text{-value}_{split}$	0.7	0.5	0.6	0.5
**Cluster radius (nm)**				
Mean ± SEM	17.1 ± 0.5	16.3 ± 0.5	13.6 ± 0.4	13.3 ± 0.2
Median	16.2	15.3	12.8	13.1
CV (%)	20.1	20.4	20.2	11.0
Skewness	1.7	1.5	2.0	0.5
Kurtosis	3.6	1.5	5.4	0.41
$P\text{-value}_{split}$	0.4	0.3	0.4	0.5
**HER2 / cluster**				
Mean ± SEM	2.7 ± 0.1	2.8 ± 0.1	2.6 ± 0.1	2.9 ± 0.1
Median	2.6	2.7	2.6	2.6
CV (%)	32.5	23.8	26.9	33.8
Skewness	1.3	0.51	1.9	0.88
Kurtosis	1.8	-0.27	6.6	0.37
$P\text{-value}_{split}$	0.4	0.5	0.6	0.5
**Clustered HER2 (%)**				
Mean ± SEM	48 ± 2	44 ± 2	34 ± 2	33 ± 1
Median	48.2	41.6	30.9	34.1
CV (%)	25.6	26.9	29.2	19.5
Skewness	0.85	0.76	0.91	-0.94
Kurtosis	0.49	0.40	0.63	1.9
$P\text{-value}_{split}$	0.6	0.7	0.5	0.3
**# of HER2 clusters / ROI**				
Mean ± SEM	1264 ± 98	928 ± 48	365 ± 22	347 ± 15
Median	967	975	315	350
CV (%)	53.3	36.6	37.6	29.9
Skewness	0.94	0.49	0.58	0.45
Kurtosis	-0.19	0.66	-0.78	-0.18
$P\text{-value}_{split}$	0.4	0.7	0.5	0.3
Coverslips imaged	3	3	3	3
Total cells	15	15	15	15
Total random ROIs	58	60	60	60
Total clustered ROIs	47	51	40	47

b	Trastuzumab-AF647		Pertuzumab-AF647	
	SK-BR-3	JIMT-1	SK-BR-3	JIMT-1
**Density (molecules/µm²)**				
Mean ± SEM	181 ± 9	22 ± 1	57 ± 3	10 ± 0.4
Median	169	22.3	50.5	9.6
CV (%)	37.3	22.9	37.3	29.0
Skewness	0.87	0.098	1.0	0.61
Kurtosis	0.021	-0.73	0.27	-0.70
$P\text{-value}_{split}$	0.5	0.5	0.2	0.6
**Cluster radius (nm)**				
Mean ± SEM	17.4 ± 0.4	14.9 ± 0.5	14.5 ± 0.4	15.8 ± 0.8
Median	16.6	14.3	13.8	14.8
CV (%)	15.3	15.3	13.7	20.6
Skewness	0.53	1.9	0.87	1.5
Kurtosis	-0.94	4.5	0.038	3.2
$P\text{-value}_{split}$	0.6	0.6	0.6	0.5
**HER2 / cluster**				
Mean ± SEM	2.9 ± 0.2	2.0 ± 0.1	2.8 ± 0.2	2.5 ± 0.2
Median	2.4	1.9	2.6	2.0
CV (%)	53.6	20.0	34.7	38.1
Skewness	2.6	0.91	1.2	0.70
Kurtosis	8.3	-0.30	1.3	-1.1
$P\text{-value}_{split}$	0.6	0.4	0.3	0.5
**Clustered HER2 (%)**				
Mean ± SEM	52 ± 2	36 ± 2	39 ± 2	39 ± 3
Median	49.3	33.4	36.9	38.6
CV (%)	19.3	23.2	23.2	32.2
Skewness	0.26	1.6	0.72	0.47
Kurtosis	-0.89	3.3	0.058	-1.0
$P\text{-value}_{split}$	0.5	0.2	0.6	0.7
**# of HER2 clusters / ROI**				
Mean ± SEM	1222 ± 84	117 ± 9	333 ± 25	62 ± 5
Median	1072	103	303	54
CV (%)	43.7	32.6	37.6	33.3
Skewness	0.80	0.88	1.4	0.64
Kurtosis	-0.38	-0.037	1.8	-0.77
$P\text{-value}_{split}$	0.4	0.8	0.6	0.5
Coverslips imaged	3	3	3	3
Total cells	15	15	14	15
Total random ROIs	60	59	55	60
Total clustered ROIs	40	20	26	17

Table S2 Summary of *p* values for qSMLM data for cultured cell lines.

	Trastuzumab-AF647		Pertuzumab-AF647		BT-474	BT-474^R	SK-BR-3	JIMT-1
	BT-474 vs. BT-474^R	SK-BR-3 vs. JIMT-1	BT-474 vs. BT-474^R	SK-BR-3 vs. JIMT-1	Trastuzumab vs. Pertuzumab	Trastuzumab vs. Pertuzumab	Trastuzumab vs. Pertuzumab	Trastuzumab vs. Pertuzumab
Mean density (molecules/μm^2)	0.003	2.4E-26	0.083	2.3E-23	4.6E-16	5.1E-21	1.1E-21	1.1E-27
Cluster radius (nm)	0.12	2.2E-04	0.30	0.071	4.1E-07	8.6E-08	1.7E-06	1.6E-01
HER2 / cluster	0.47	7.4E-04	0.097	0.25	0.26	0.25	0.31	0.026
Clustered HER2 (%)	0.039	1.7E-08	0.32	0.50	2.3E-08	1.1E-07	7.2E-07	0.18
# of HER2 clusters / ROI	0.002	3.2E-16	0.24	1.3E-11	2.8E-12	2.4E-17	1.7E-13	2.6E-06

Table S3. Patient characteristics.

	P18	P20	P21	P23	P25	P26
Age	50	59	31	63	61	56
Pre-op clinical stage	T1 N1	T2 N0	T4 N1	T2 N0	T2 N1	T2 N1
ER (biopsy)	negative	positive	positive	positive	positive	positive
PR (biopsy)	negative	negative	negative	positive	positive	negative
HER2 IHC (biopsy)	3+	2+	3+	3+	3+	3+
HER2 FISH copy number (biopsy)	N/A	2	18.8	32.7	N/A	15.5
HER2 FISH ratio (biopsy)	N/A	1	9.2	10.1	N/A	11.1
Ki-67 (biopsy)	N/A	75%	N/A	20%	50%	70%
Post-op pathologic stage	T1a N0	T1b N1a	T0 N0	T0 N0	T0 N0	Tis N0
RCB	RCB-I	RCB-II	pCR	pCR	pCR	pCR

Table S4 Summary of qSMLM data for patient samples.

	P18	P20	P21	P23	P25 S1	P25 S2	P26
**Density (molecules/μm2)**							
Mean ± SEM	49 ± 7	58 ± 4	123 ± 7	157 ± 7	99 ± 7	137 ± 9	59 ± 6
Median	34.5	47.5	101	136	104	120	47.2
CV (%)	95.5	53.9	44.3	46.6	46.8	50.2	79.1
Skewness	2.2	0.55	0.6	0.57	-0.074	0.43	1.4
Kurtosis	4.3	-0.91	-0.064	-0.84	-1.1	-0.75	1.5
P-valuesplit	0.5	0.5	0.5	0.6	0.5	0.5	0.3
**Cluster radius (nm)**							
Mean ± SEM	18 ± 1.8	26 ± 1.5	34 ± 2.2	25 ± 1.1	34 ± 5.1	31 ± 1.3	38 ± 6.3
Median	15	25.8	29.9	22.1	28.3	30	30.1
CV (%)	40.3	11.2	49.9	38.3	45.1	18.9	49.7
Skewness	2.5	0.3	2.5	1.4	1.4	0.78	1.7
Kurtosis	6.5	-0.35	8	1.4	2.5	0.097	2.8
P-valuesplit	0.3	0.4	0.6	0.8	0.5	0.4	0.6
**HER2 / cluster**							
Mean ± SEM	2.3 ± 0.2	1.8 ± 0.1	3.8 ± 0.3	2.7 ± 0.1	3.0 ± 0.7	2.6 ± 0.1	2.9 ± 0.5
Median	2	1.78	3.18	2.37	2.06	2.43	2.31
CV (%)	38.6	6.03	69.9	38.4	73.8	22.7	54.2
Skewness	3.1	-0.34	3.7	1.3	2.5	0.39	1.5
Kurtosis	11.2	-4	16	1.3	6.5	-1.1	1.5
P-valuesplit	0.3	0.4	0.7	0.7	0.5	0.6	0.4
**Clustered HER2 (%)**							
Mean ± SEM	33 ± 4	31 ± 2	64 ± 2	54 ± 2	66 ± 7	59 ± 2	73 ± 5
Median	32.2	29.8	63.8	51.2	62	58.2	73.3
CV (%)	48	11.3	28.9	34.4	31.3	18.2	21.6
Skewness	1.8	1.2	-0.016	0.73	-0.052	0.042	-0.0057
Kurtosis	4	0.74	-0.91	-0.29	-1.6	-0.33	-1.7
P-valuesplit	0.6	0.6	0.4	0.4	0.2	0.6	0.5
**# of HER2 clusters / ROI**							
Mean ± SEM	199 ± 51	118 ± 33	732 ± 49	878 ± 60	909 ± 82	778 ± 71	638 ± 89
Median	145	99	604	690	910	651	625
CV (%)	105	55.8	51.9	57.5	27.2	41.7	41.8
Skewness	2	1.3	0.7	0.87	0.65	1.1	0.92
Kurtosis	3.2	1.2	-0.4	0.17	-0.69	0.72	2.2
P-valuesplit	0.7	0.5	0.6	0.5	0.5	0.7	0.5
Coverslips Imaged	2	2	2	2	2	2	2
Total cells	12	14	20	21	14	15	14
Total random ROIs	47	58	69	102	45	60	56
Total clustered ROIs	17	4	61	70	9	21	9

Table S5 Summary of *p* values for qSMLM data for patient samples.

		Mean density (molecules/μm²)	Cluster radius (nm)	HER2 / cluster	Clustered HER2 (%)	# of HER2 clusters / ROI
P18 vs.	P20	0.15	0.002	0.017	0.31	0.099
	P21	7.8E-12	1.8E-07	1.6E-04	5.5E-08	4.8E-10
	P23	2.5E-19	0.006	0.037	3.4E-05	1.4E-12
	P25 S1	1.3E-06	0.007	0.19	5.0E-04	1.7E-06
	P25 S2	4.5E-12	1.1E-06	0.13	2.1E-06	5.9E-08
	P26	0.14	0.006	0.13	6.3E-06	4.1E-04
P20 vs.	P21	6.5E-14	0.003	8.8E-08	9.2E-11	7.9E-11
	P23	9.2E-24	0.047	1.3E-09	8.3E-08	8.9E-14
	P25 S1	9.5E-07	0.084	0.068	3.9E-04	2.1E-06
	P25 S2	2.0E-12	0.021	3.8E-06	2.7E-08	9.5E-09
	P26	0.41	0.047	0.029	1.2E-05	1.4E-04
P21 vs.	P23	3.1E-04	0.27	0.002	6.5E-04	0.031
	P25 S1	0.007	0.50	0.17	0.40	0.042
	P25 S2	0.10	0.099	5.7E-04	0.048	0.30
	P26	7.0E-11	0.27	0.10	0.088	0.19
P23 vs.	P25 S1	3.0E-08	0.30	0.37	0.057	0.38
	P25 S2	0.042	0.14	0.16	0.061	0.14
	P26	3.2E-19	0.50	0.35	0.003	0.020
P25 S1 vs.	P25 S2	5.6E-04	0.27	0.29	0.16	0.12
	P26	2.2E-05	0.30	0.49	0.24	0.020
P25 S2 vs.	P26	6.2E-11	0.14	0.24	0.016	0.12
